# Identifying key outcome domains with underlying specific patient-reported outcomes for psychomotor therapy in mental health care in the Netherlands: a multi-phased qualitative study

**DOI:** 10.1007/s11136-025-04119-2

**Published:** 2026-01-21

**Authors:** Albertine de Haan, Janet Moeijes, Mia Scheffers, Philip van der Wees

**Affiliations:** 1 Department of Human Movement and Education, Windesheim University of Applied Sciences, Zwolle, the Netherlands; 2 Radboud University Medical Center, IQ Health and Department of Rehabilitation, Nijmegen, the Netherlands

**Keywords:** Patient-reported outcomes, Mental health, Psychomotor therapy, Patient-reported outcome measures

## Abstract

**Purpose:**

Psychomotor therapy is an experiential therapy using movement- and body-oriented interventions to diminish psychiatric symptoms and improve psychosocial functioning. However, routine evaluation of patient-reported outcomes (PROs) and standardisation of patient-reported outcome measures (PROMs) in psychomotor therapy research and practice in adult mental healthcare are lacking, resulting in a gap in systematic research and evaluation of psychomotor interventions. This study aims to select the five most relevant outcome domains with underlying PROs for psychomotor therapy from the perspective of psychomotor professionals and patients.

**Method:**

A multi-phased qualitative study was conducted in the Netherlands, consisting of three sub-studies: (i) the selection of the five most relevant outcome domains with underlying PROs from the perspective of psychomotor professionals (N = 53), using a modified Nominal Group Technique in an adapted serial design; (ii) the selection of the five most relevant outcome domains with underlying PROs from the perspective of patients (N = 27) using a narrative approach in (focus) groups; and (iii) the synthesised selection from (i) and (ii).

**Results:**

Psychomotor professionals selected body experience, movement experience, emotion regulation, stress regulation, and sensory awareness as the most relevant outcome domains. Patients selected body experience, social interaction, movement experience, emotion regulation, and integration of thinking, feeling and behaviour. After synthesising both selections, the five most relevant outcome domains for psychomotor therapy are body experience, movement experience, emotion regulation, social interaction, and stress regulation.

**Conclusion:**

The five most relevant outcome domains with underlying PROs in psychomotor therapy in adult mental healthcare in the Netherlands have been identified and are broadly supported by psychomotor professionals and patients. These outcome domains provide the foundation for selecting PROMs for evaluating interventions and monitoring outcomes in psychomotor therapy.

**Supplementary Information:**

The online version contains supplementary material available at 10.1007/s11136-025-04119-2.

## Introduction

Psychomotor therapy is an experiential therapy making use of movement and body-oriented interventions to diminish psychiatric symptoms, increase mental health, and improve psychosocial functioning [1]. Psychomotor therapy is widely used in mental healthcare treatments in the Netherlands, suitable for patients with a wide range of psychopathology [2] and highly valued by patients [3]. However, evidence about the effectiveness of psychomotor interventions and insight into outcomes that patients report is sparse [4].

Currently, Patient-Reported Outcome Measures (PROMs) are successfully used in mental health care [5]. PROMs are self-administered questionnaires for patients consisting of one or multiple items to assess Patient-Reported Outcomes (PROs). PROs are aspects of a patient’s health status directly reported by the patient without interpretation of the patient’s response by a clinician, such as feeling anxious when meeting new people or feeling disconnected from one’s own body. PROs are subjective aspects of one’s health, in contrast to objective measures like physiological aspects, e.g. blood pressure. PROs can be clustered into outcome domains specific to health-related constructs, e.g. a function (e.g. sleep), a disease (e.g. anxiety disorder) or a symptom (e.g. impulsivity). PROMs with multiple items may measure one or more domains consisting of several specific PROs [6–8].

PROMs can be used to provide insight into the health outcomes of patients, accumulate evidence for interventions, and contribute to comparing the effectiveness of interventions [9]. Besides these research purposes, PROMs are increasingly used in daily practice at an individual level for assessment, clinical decision-making, and tracking and evaluating individual treatment results [10].

According to a recent meta-analysis [11], the beneficial effects of the use of PROMs in mental healthcare can include symptom reduction and a decrease in dropouts. Moreover, discussing PROM results in psychotherapy may foster greater patient self-awareness and self-reflection [12]. Therefore, applying PROMs in psychomotor therapy to evaluate interventions and monitor individual patient outcomes constitutes a logical next step.

Most PROMs in mental healthcare obtain generic outcomes concerning mental health problems, such as depression and not specific outcomes for psychomotor therapy. Although psychomotor therapy is increasingly evaluated in research with PROMs that measure specific outcomes for psychomotor therapy, such as body experience [13–15] and emotion regulation [16], the amount of psychomotor research is still limited. Some mental health institutes employ promising PROMs to assess specific outcomes for psychomotor therapy, such as the Multidimensional Assessment of Interoceptive Awareness-2 (MAIA-2) [17], which measures body awareness. However, a standardised use of PROMs for specific outcomes for psychomotor therapy in research and psychomotor practice is lacking, resulting in a gap in systematic research and evaluation of psychomotor interventions. To date, the outcome domains and PROs relevant for psychomotor therapy, as identified by professionals and patients, and corresponding PROMs for their assessment, remain undefined. No studies have yet determined the most relevant outcome domains for psychomotor therapy in adult mental health care in the Netherlands. Establishing these domains will provide a foundation for selecting PROMs that can measure them with precision and reliability—an essential step for systematically evaluating psychomotor therapy at both the individual and research levels.

This study aimed to identify the five most relevant outcome domains with underlying specific PROs in the field of psychomotor therapy. This prioritisation was based on pragmatic considerations to avoid an excessive proliferation of PROMs to measure outcomes in psychomotor therapy.

The research question is: Which five outcome domains with underlying specific PROs for psychomotor therapy are most relevant from a synthesised perspective of psychomotor professionals and patients in adult mental healthcare in the Netherlands?

## Methods

This study was reported in accordance with the ACCORD (ACcurate COnsensus Reporting Document) guidelines.

The methodology presents a concise overview of the study’s key procedures. The methodology of the study is presented in detail in Online Resource 2, thereby promoting both transparency and the interpretability of the findings.

### Design and setting

This study, conducted from September 2019 to February 2020, employed a qualitative multi-phased approach. It consisted of three sub-studies:

(i) the identification and prioritisation of the most relevant outcome domains with underlying specific PROs for psychomotor therapy from the perspective of psychomotor professionals with the use of a modified Nominal Group Technique in an adapted serial design (NGT) [18-20];
(ii) the identification and prioritisation of the most relevant outcome domains with underlying specific PROs for psychomotor therapy from the perspective of patients using a narrative approach [21] in focus groups; and,
(iii) the synthesis of the priority of both selections, resulting in the five most relevant outcome domains with underlying specific PROs for psychomotor therapy.

**Fig. 1:**
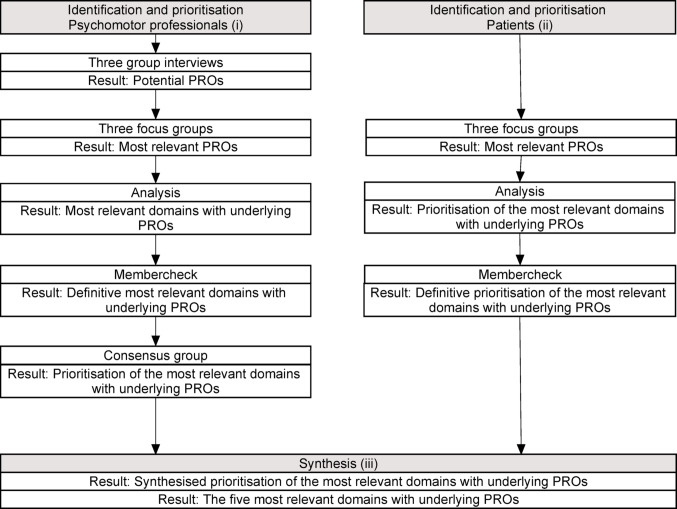
Flowchart of sub-studies (i)–(iii) depicting each step with the procedure performed, and the corresponding results. *Note*: Made with ClickCharts NCH Software

Figure 1 shows the steps in the sub-studies. Each step in the figure shows the procedures performed and the corresponding results

To enhance the comprehensiveness and clarity of the five most relevant outcome domains with underlying PROs for psychomotor therapy, the domains were categorised using the International Classification of Functioning, Disability and Health (ICF) developed by the World Health Organization [22, 23]. Methods for linking the outcome domains to the ICF framework are described in Online Resource 1.

### Sub-study (i): identification and prioritisation of the most relevant outcome domains with underlying specific pros for psychomotor therapy by psychomotor professionals

A classical NGT face-to-face group meeting consists of solo idea generation, round-robin feedback on ideas, clarification, and voting to prioritise ideas [24]. In this sub-study the modified NGT was applied across three group interviews (idea generation of PROs), three focus groups (individual selection of the most relevant PROs and clarification), and one consensus group meeting (voting to prioritise domains with underlying PROs). Due to the inclusion of the total number of groups and diverse participants in the study, data saturation [25, 26] was anticipated.

#### Group interviews

In three group interviews, semi-structured interviews conducted with several people at the same time [27], we used the first step of the modified NGT to generate potential PROs for psychomotor therapy in adult mental healthcare.

#### Participants

The three groups consisted of lecturers of all initial and master’s educational programs in psychomotor therapy in the Netherlands. Participation was based on availability. Lecturers were affiliated with the bachelor’s degree (group 1; *n* = 20) and master’s degree (group 2; *n* = 6) programs in psychomotor therapy at Windesheim University of Applied Sciences, Zwolle and the bachelor’s degree (group 3; *n* = 5) program at HAN University of Applied Sciences, Nijmegen.

#### Data collection

In 60-minute group interviews, participants were asked to identify potential PROs for psychomotor therapy in adult mental health care and to elaborate on what each PRO entailed.

#### Data analysis

Outcomes that were not considered PROs (objective measures, i.e., physical fitness) were excluded [7, 28]. Finally, a list of potential PROs for psychomotor therapy in adult mental healthcare was compiled.

#### Focus groups

In three focus groups, guided group discussions to explore participants’ perspectives through interaction [29], the second step of the modified NGT was used to select the most relevant PROs for psychomotor therapy.

#### Participants

Three distinct focus groups were selected through the purposive sampling of different psychomotor professionals to ensure diverse perspectives. Participants were researchers in psychomotor therapy of the research group Human Movement, Health, and Well-being at Windesheim University of Applied Sciences Zwolle (group 1; *n* = 7), psychomotor therapists from the University Centre of Psychiatry (UCP) at University Medical Centre Groningen (UMCG; group 2; *n* = 6), and a group of senior psychomotor therapists selected through quota sampling [30] based on expertise in different psychopathology (group 3; *n* = 10). One person participated in both groups because of extensive expertise.

#### Data collection

During three 90-minute focus groups, participants were provided with sets of index cards, each displaying a single PRO previously identified in the group interviews. In the first phase, participants independently selected the five PROs they considered most relevant, without discussion, by choosing the corresponding index cards. Participants were also allowed to introduce new PROs if they identified gaps. In the second phase, participants shared their selections in a group discussion to explore underlying rationales. In the final phase, participants selected their final five most relevant PROs for psychomotor therapy.

#### Data analysis

After each focus group, newly mentioned PROs were added to the index cards for the next meeting. PROs were clustered and assigned to outcome domains by two researchers.

Finally, a report of the most relevant outcome domains with underlying specific PROs for psychomotor therapy was compiled and sent to all participants of the group interviews and focus groups for a member check. The participants’ feedback was processed (AH, JM, in case of non-agreement MS was consulted), resulting in a definitive report.

#### Consensus group meeting

In a consensus group meeting, the third and last step of the modified NGT was used to prioritise the most relevant outcome domains with underlying specific PROs for psychomotor therapy.

#### Participants

A 90-minute consensus group meeting (*n* = 6) included participants from the previous group interviews and focus group meetings to ensure representation from the groups based on willingness and availability.

#### Data collection and data analysis

A week before the meeting, participants received the definitive report of the results of the focus groups. All voting was individual, subjective and anonymous by using Mentimeter [31].

Each participant assigned the highest rank to the domain they considered most relevant, followed by successive ranks in descending order of relevance, with the lowest rank assigned to the domain judged least relevant. Mentimeter automatically calculated the mean ranking for each domain across participants and subsequently generated an aggregated list. In this list, the domain with the highest mean rank reflected greater perceived relevance as determined by the participants and received the highest ranking number.

In a subsequent step, participants distributed a total of 100 points (in total 600 points by all participants) across all domains using Mentimeter. A higher number of points indicated greater perceived relevance, whereas fewer or zero points reflected lesser relevance. Mentimeter then produced an aggregated list ordered from the highest to lowest points received, with the domain receiving the most points at the top of the list, being perceived as the most relevant, receiving the highest rating number.

For the analysis, the ranking and rating numbers of the domains from both lists were summed, with lower totals indicating higher relevance. In the event of ties, the number of times a domain was selected in the focus group determined its priority, with more frequently selected domains considered more relevant. Then, the researchers produced an aggregated list of the domains in order of relevance.

Finally, the participants rated their percentage of agreement (0-100%) with the order of relevance of the domains, using Mentimeter independently and subjectively. Consensus was considered at a mean of 70% agreement [19]. In case of less than 70% agreement, a group discussion, rating, ranking and consensus rating were repeated, until 70% agreement was reached.

A list was compiled (AH, JM), presenting the prioritisation of the most relevant outcome domains with their underlying specific PROs for psychomotor therapy, as identified by psychomotor professionals.

### Sub-study (ii): identification and prioritisation of the most relevant outcome domains with underlying specific pros for psychomotor therapy by patients

To identify and prioritise the most relevant domains and underlying specific PROs for psychomotor therapy from the patient’s perspective, three 90-minute face-to-face focus groups with a narrative approach were conducted.

#### Participants

Participants were recruited in collaboration with the mental health institute Altrecht in Utrecht (group 1; *n* = 12) and the UCP of the UMCG Groningen (group 2; *n* = 8 and group 3; *n* = 7). Participants at the UCP were assigned to a focus group based on their availability. Purposive sampling was used to invite patients with different psychiatric disorders who had undergone group- and/or individual psychomotor treatment. Inclusion criteria were the ability to engage in a group conversation and be older than 18 years of age.

#### Data collection

In the patient focus groups, each participant was asked to identify and select their three most relevant PROs from their individual experience, rather than five, as in the professional group, since patients primarily focus on their own experiences. In contrast, psychomotor professionals draw on broader clinical experience across diverse patients and therefore had the opportunity to incorporate their knowledge in those five PROs.

#### Data analysis

Prioritisation of domains with underlying specific PROs for psychomotor therapy was based on the number of selected PROs in a domain, with higher prevalence indicating higher prioritisation. A report of the prioritised outcome domains with underlying specific PROs for psychomotor therapy was sent to all participants for a member check. The participants’ feedback was processed, resulting in a definitive report on the prioritisation of the most relevant domains with underlying specific PROs for psychomotor therapy from the patient’s perspective.

### Sub-study (iii) the synthesis of the priority of sub-study (i) and sub-study (ii), resulting in the five most relevant outcome domains with underlying specific pros for psychomotor therapy

To synthesise the prioritisations from the perspective of psychomotor professionals (i) and patients (ii), and to identify the five most relevant outcome domains, the research team held a 90-minute meeting.

#### Participants

All members of the research team (*n* = 6) participated in the meeting.

#### Data analysis

First, the executing researchers presented the results. Domains from both studies were merged where appropriate.

Then, the prioritisations of the most relevant domains of both psychomotor professionals and patients were listed in order of relevance. The orders of relevance of the domains from both lists were summed and divided by two (or one if a domain was only mentioned by one group) to calculate the mean sum of the orders of relevance, with lower totals indicating higher relevance. As a result, the synthesised prioritisation of the most relevant domains with underlying specific PROs for psychomotor therapy was compiled.

## Results

The results of the multi-phased qualitative study to find the five most relevant outcome domains with underlying specific PROs for psychomotor therapy are displayed in line with the sub-studies.

### Sub-study (i): identification and prioritisation of the most relevant outcome domains with underlying specific pros for psychomotor therapy by psychomotor professionals

The characteristics of the participants of sub-study (i) are shown in Table 1.

**Table 1:** Characteristics of psychomotor professionals (*n* = 53)^1^ in substudy (i)

	Group interviews (*n* = 31)	Focus groups (*n* = 23)	Consensus meeting (*n* = 6)
*Gender*			
Male	-	10	3
Female	-	13	3
*Profession* ^*2*^			
Psychomotor therapist	-	18	2
Research	-	9	2
Education	-	4	4
*Educational program* ^*3*^			
Bachelor Windesheim University	20	2	1
Master Windesheim University	6	3	3
Bachelor HAN University	5	0	1

^1^ The total number of psychomotor professionals is less than the sum of all groups due to participation in more than one group of some psychomotor professionals

^2^ The total number of professions exceeds the total number of psychomotor professionals due to the various professions of some psychomotor professionals

^3^ The total number of educational programs exceeds the total number of psychomotor professionals due to the various educational activities of some psychomotor professionals

Collected PROs that referred to general outcomes, such as well-being, and outcomes that could not be measured with PROMs, such as physiological outcomes (i.e. physical fitness) were excluded from the analysis. A total of 14 new PROs were identified in the focus groups. Although a single new theme was identified in the concluding focus group, the research team determined that data saturation had been reached, given that no further meaningful insights were anticipated

Based on the member check of the report, self-efficacy was placed from the domain of movement experience to the domain of self-regulation. The final report for the consensus group identified nine outcome domains with 38 underlying specific PROs for psychomotor therapy

In the consensus group, the participants rated their agreement with the first prioritisation of the most relevant domains at a mean of 80%

Table 2 presents the most relevant outcome domains and subdomains with underlying specific PROs for psychomotor therapy in order of relevance as determined by psychomotor professionals, accompanied by the number of selections in the focus groups, the ranking numbers, rating numbers and the sum of the ranking and rating numbers in the consensus group.

**Table 2:** The most relevant outcome domains and subdomains with underlying specific pros for psychomotor therapy, according to psychomotor professionals, in order of relevance, along with the number of focus group selections, the ranking, the rating, and the sum of the last two

Outcome domain	Subdomain	PRO	Focus group (*n* = 23)	Consensus group (*n* = 6)		
			Selection (*n*)	Ranking number (M)	Rating number	Sum of ranking and rating number
(1) Body experience	Body awareness	Awareness of bodily signals Recognising bodily signals Meaning of bodily signals Feeling boundaries Trust in one’s own body	25	1	1	2
	Body attitude	Behaviours, thoughts, and feelings in relation to one’s own body				
	Body satisfaction	Satisfaction with the body’s shape or appearance Body image				
(2) Movement experience	Enjoyment in physical activity	Having a good time when playing sports or going to the gym Playfulness	9	2	2	4
	Motivation to be physically active	Being able to be physically active when one wants to Activation				
	Perceived competence in being physically active	Thoughts about one’s own movement qualities Experiencing one’s strength				
(3) Emotion regulation		Recognising emotions Expressing emotions Impulse regulation Coping with emotions Accepting emotions	19	3	3	6
(4) Stress regulation		Coping effectively with stress and arousal Relaxation	10	5	4	9
(5) Sensory awareness		Awareness of the information from the physical senses Listening to the body’s information	9	4	5	9
(6) Social interaction		Social competence Social skills Relational skills Empathy Assertiveness	5	6	6	12
(7) Integration of thinking, feeling and behaviour		Self-expression Mind-body integration Taking one’s own feelings seriously Autonomy	24	8	6	14
(8) Self-regulation		Control of behaviour Flexibility of one’s own behaviour Self-efficacy	8	9	6	15
(9) Body empowerment		Vitality Empowerment Strength	6	6	9	15

Selection (N) refers to the number of selections of an outcome domain in the focus groups

Ranking number (M) refers to the mean ranked place of the ranking in order of relevance, of all the outcome domains by all participants of the consensus group, with a lower number indicating higher relevance

Rating number refers to the rated place assigned to an outcome domain by all participants of the consensus group, with a lower number receiving more points and indicating a higher relevance

Sum of ranking and rating numbers refers to the sum of ranking and the rating numbers of the outcome domains, with a lower number indicating higher relevance

### Sub-study (ii): Identification and prioritisation of the most relevant outcome domains with underlying specific PROs for psychomotor therapy by patients

The study involved a total of 27 patients aged 18 years and older (9 men (33.3%) and 18 women (66.7%)). These patients were recruited from two different healthcare institutions, UMCG (4 men, 11 women) and Altrecht (5 men, 7 women). All participants had undergone group- or individual psychomotor therapy. Participants were diagnosed with various disorders, including personality disorders, anxiety disorders, mood disorders, posttraumatic stress disorder, and eating disorders

After conducting three focus groups, no additional PROs for psychomotor therapy were identified, indicating saturation had been reached. Based on the member check of the report, trust in one’s own body was added as a PRO to the domain of body experience. Also, the balance between being physically active and resting was added as a PRO to the domain of movement experience. The final report identified eight outcome domains with 28 underlying specific PROs for psychomotor therapy

Table 3 displays the most relevant outcome domains with underlying specific PROs for psychomotor therapy as identified and prioritised by patients, along with patient quotes, and the total number of selections of PROs in the domain, in order of relevance.

**Table 3:** The most relevant outcome domains with underlying specific pros for psychomotor therapy as identified and prioritised by patients (*n* = 27), in order of relevance, along with patient quotes, and the total number of selections of pros in the domain

Outcome domain	PRO	Quote	Selection (*n*)
(1) Body experience	Meaning of bodily signals Awareness of bodily signals Body image Listening to one’s body Feeling safe in one’s body Feeling the boundaries of one’s body	‘My body reacts before my brain does.’	22
(2) Social interaction	Experiencing boundaries in interaction Awareness of social interaction Being able to share experiences with others Feeling connected with others Asking for help Feeling safe in social interaction	‘I learned how to stay and feel connected with myself and others in a group.’	19
(3) Movement experience	Activation Feeling one’s strength Enjoyment in physical activity The balance between being physically active and resting	‘I appreciated receiving support to motivate myself to be active. I love to be physically active, but starting up is sometimes difficult.’	10
(4) Emotion regulation	Feeling emotions Expressing emotions Coping with emotions	‘Because of psychomotor therapy, I can feel more emotions.’	9
(5) Integration of thinking, feeling and behaviour	Connection between body and mind Being more aware of the meaning of one’s own movement behaviour.	‘My mind and my body are more connected to each other.’	8
(6) Self- image	Self-acceptance Self-confidence Realistic self-image	‘With the help of psychomotor therapy, I learned to be less demanding of myself and I got to know my possibilities.’	7
(7) Stress regulation	Feeling balanced between being stressed and being relaxed Feeling less stressed	‘I learned how to relax my body in psychomotor therapy.’	3
(8) Sensory attention	Being able to look at something without judgment Less dissociation	‘At psychomotor therapy, I learned to dissociate less by sitting on a ball and moving calmly, I stayed with my attention in the gym.’	2

Selection (N) refers to the number of selections of an outcome domain in the focus groups

### Sub-study (iii) The synthesis of the priority of sub-study (i) and sub-study (ii), resulting in the five most relevant outcome domains with underlying specific PROs for psychomotor therapy

In the synthesis, the outcome domains of sensory awareness and sensory attention were merged with the outcome domain of body experience since sensory awareness and sensory attention can be considered key elements of body awareness, a component of body experience [32]. The outcome domain of integration of thinking, feeling, and behaviour was also excluded from the synthesis, as it may be regarded as a multitheoretical framework for psychotherapy [33], a framework that is likewise applied in psychomotor therapy [1]. The construct of this framework is a multidimensional concept that is challenging to measure with a single PROM, despite extensive research efforts [34]. The synthesis identified eight outcome domains with 48 underlying specific PROs for psychomotor therapy.

In Table 4, the most relevant outcome domains, subdomains with underlying specific PROs for psychomotor therapy, in order of relevance from both perspectives, the order of relevance of the domains from psychomotor professionals and patients, and the mean sum of the order of relevance.

**Table 4:** The most relevant outcome domains, subdomains with underlying specific pros for psychomotor therapy, in order of relevance from both perspectives, the order of relevance of the domains from psychomotor professionals and patients and the mean sum of the orders of relevance of both

Outcome domain	Subdomain	PRO	Order of relevance psychomotor professionals	Order of relevance patients	Mean score of the orders of relevance
(1) Body experience	Body awareness	Awareness of bodily signals (including sensory awareness) Recognising bodily signals Meaning of bodily signals Listening to bodily signals Feeling the boundaries of one’s own body Trust in one’s own body Feeling safe in one’s body Sensory attention	1	1	1
	Body attitude	Behaviours, thoughts, and feelings in relation to one’s own body			
	Body satisfaction	Satisfaction with the body’s shape or appearance Body image			
(2) Movement experience	Enjoyment in physical activity	Having a good time when playing sports or going to the gym Playfulness	2	3	2,5
	Motivation to be physically active	Being able to be physically active when one wants to Activation The balance between being physically active and resting			
	Perceived competence in being physically active	Thoughts about one’s own movement qualities Experiencing one’s strength			
(3) Emotion regulation		Feeling emotions Recognising emotions Expressing emotions Impulse regulation Coping with emotions Accepting emotions	3	4	3,5
(4) Social interaction		Social competence Social skills Relational skills Empathy Assertiveness Experiencing boundaries in interaction Awareness of social interaction Being able to share experiences with others Feeling connected with others Asking for help Feeling safe in social interaction	6	2	4
(5) Stress regulation		Relaxation Coping effectively with stress and arousal Feeling less stressed Feeling balanced between being stressed and being relaxed	4	7	5,5
(6) Self-image		Self-acceptance Self-confidence Realistic self-image		6	6
(7) Self-regulation		Control of behaviour Flexibility of one’s own behaviour Self-efficacy	8		8
(8) Body empowerment		Vitality Empowerment Strength	9		9

Mean sum of the order of relevance refers to mean sum of the orders of relevance from sub-study (i) and (ii), with lower totals indicating higher relevance

After conducting the synthesis, we identified the five most relevant outcome domains from the perspective of psychomotor professionals and patients which are body experience, movement experience, emotion regulation, social interaction and stress regulation.

### ICF framework

The results of linking the five most relevant outcome domains for psychomotor therapy to the ICF framework are reported in Online Resource 1. The identified outcomes domains could be linked to the ICF domains of mental functions, sensory functions and pain, learning and applying knowledge, general tasks and demands, mobility, community, social and civic life, communication, interpersonal interaction, and relationships.

## Discussion

This study is the first to prioritise the five most relevant outcome domains with underlying specific PROs for psychomotor therapy from the perspective of psychomotor professionals and patients in adult mental healthcare. The selected five most relevant outcome domains for psychomotor therapy are body experience, emotion regulation, movement experience, social interaction and stress regulation. Both psychomotor professionals and patients considered body experience, emotion regulation, and movement experience to be among the five most relevant domains for psychomotor therapy. Some differences emerged in prioritisation between psychomotor professionals and patients: psychomotor professionals selected sensory awareness, whereas patients selected social interaction and the integration of thinking, feeling and behaviour. Sensory awareness was merged with body experience. The domain of integration of thinking, feeling, and behaviour was excluded.

The five most relevant outcome domains for psychomotor therapy, as identified in this study, are of significant importance. They have already been used in psychomotor therapy research with outcomes defined as body experience [13–15], emotion regulation [16,35] and stress regulation [35, 36]. However, the amount of psychomotor therapy research using these five outcome domains is still scarce. Due to this limitation, these domains have not been consistently reported in systematic reviews on psychomotor and body- and movement-oriented therapies in mental healthcare [37]. Systematic reviews predominantly report and evaluate generic psychiatric symptomatology measures [37–40]. Although Rosendahl et al. [37] analysed outcomes related to body experience and interpersonal problems (outcome domain: social interaction), and van de Kamp et al. [40] analysed interoception (outcome domain: body experience), the number of studies included in these analyses was limited, and the concepts measured were diverse, contributing to considerable heterogeneity in the findings [37,40]. These observations underscore the need for greater consistency in defining and measuring the five most relevant outcome domains for psychomotor therapy.

It is worth mentioning that the identified five most relevant outcome domains for psychomotor therapy may be considered transdiagnostic factors in psychopathology. Transdiagnostic factors are underlying psychological, cognitive, behavioural, and physiological mechanisms contributing to the development and maintenance of various psychiatric disorders [41–43]. Disturbances in body experience are implicated in various psychiatric disorders, including mood disorders [44], trauma-related disorders [45], somatoform disorders [46], and chronic pain [15]. Furthermore, emotion regulation is a key factor in many psychopathologies [41, 47, 48] and is linked to stress regulation and stress-related coping [49, 50]. Additionally, interpersonal dysfunction is prevalent across various psychiatric disorders [51–53]. As anhedonia [54], self-perceived competence [55,56], and motivation [57] are associated with multiple psychiatric disorders, disturbances in movement pleasure, competence and motivation may also be relevant across disorders. Thus, the selected five outcome domains apply to various groups of patients receiving psychomotor treatment.

The five most relevant outcome domains for psychomotor therapy identified in this study were categorised using the ICF model [23]. Two previous publications on psychomotor therapy for chronic pain management [58] and a psychomotor observation tool for children and adolescents [59] also linked their psychomotor goals and observation domains to the ICF model. Both studies selected similar ICF domains to this study: mental functions, learning and applying knowledge, general tasks and demands, mobility, community, civic and social life, communication, and interpersonal interactions. Emck et al. [59] also included sensory functions and self-care, consistent with this study.

The strengths of this study include the use of standardised methods, triangulation, and data saturation in both data collection and analysis. Additionally, debriefing and member checking enhanced the study's dependability and trustworthiness. Specifically, using NGT diminished the influence of peer pressure on individuals in a group to form a consensus [60]. Also, separate groups of psychomotor professionals and patients were formed to minimise ‘power differentials’ between participants [24]. Another strength of this study is the participation of both psychomotor professionals and patients with diverse expertise in adult mental healthcare, which provided a comprehensive perspective on the outcomes of psychomotor therapy.

Although psychomotor professionals and patients with various expertise in psychiatric disorders participated in the study, specific records of participant characteristics are absent. This limitation may influence the transferability of the results. Another limitation may be the potential selection bias of the study participants. Participation was based on availability and willingness to participate, and specific records of patients are not available. Although we aimed for the maximum possible number of participants and sought an even distribution of expertise within and across groups, this may still have influenced the results. The potential impact of the working relationship between the researchers and some psychomotor professionals was another limitation of this study. The individual generation and selection of ideas in the NGT method minimised this potential effect. Moreover, the impact of the working relationship was continuously discussed during debriefing sessions.

The insights of this study facilitate the selection of PROMs to assess the identified five most relevant outcome domains with their underlying specific PROs for psychomotor therapy, as well as the systematic evaluation of psychomotor therapy both on a patient level and for research objectives. Further development in (digital) measurement systems for psychomotor therapists and patients, and accessibility to those systems, is necessary to enhance the use of PROMs in psychomotor therapy. Appropriate policy support at the institute level is desired to support the implementation and the use of PROMs.

Another promising line of research would be to conduct similar research for other psychomotor domains in the Netherlands, e.g. child and adolescent psychiatry and rehabilitation. Besides, comparable research in other countries can enhance international collaboration on psychomotor therapy research. This study’s methodology may provide a guideline for designing such studies.

In conclusion, according to psychomotor professionals and patients, the five most relevant outcome domains in psychomotor therapy in adult mental healthcare in the Netherlands are body experience, emotion regulation, movement experience, social interaction, and stress regulation. These domains with underlying specific PROs for psychomotor therapy are crucial for the systematic evaluation of psychomotor therapy both on an individual level and for research objectives. The next step is to find corresponding PROMs followed by an implementation study of the use of PROMs in psychomotor therapy practices.

## Supplementary Information

Below is the link to the electronic supplementary material.

Supplementary Material 1

Supplementary Material 2

## References

[1] Emck, C., & Scheffers, M. (2019). Psychomotor interventions for mental health: An introduction. In J. de Lange, O. Glas, J. van Busschbach, C. Emck, & T. Scheewe (Eds.). *Psychomotor interventions for mental health-Adults: A movement-and body-oriented approach* (pp. 17–51). Boom.

[2] de Lange, J., Glas, O., Van Busschbach, J., Emck, C., & Scheewe, T. (2019). *Psychomotor interventions for mental health-Adults: A movement-and body-oriented approach*. Boom.

[3] Meijnckens, D., & Hesselink, A. (2016). *Achterbanraadpleging Zorgstandaard trauma- en stressorgerelateerde stoornissen [Constituency consultation Care standard trauma and stressor-related disorders]*. LPGGz.

[4] Borgesius, E., & Visser, E. C. M. (2015). *Rapport vaktherapie en dagbesteding in de geneeskundige GGZ [Report on experiental therapy and day care in the medical mental health system].* Zorginstituut Nederland.

[5] Kendrick, T., El-Gohary, M., Stuart, B., Gilbody, S., Churchill, R., Aiken, L., Bhattacharya, A., Gimson, A., Brütt, C., De Jong, K., & Moore, M. (2016). Routine use of patient-reported outcome measures (PROMs) for improving treatment of common mental health disorders in adults. *Cochrane Database of Systematic Reviews*, *7*(7). 10.1002/14651858.CD011119.pub2 10.1002/14651858.CD011119.pub2 PMC6472430 27409972

[6] Churruca, K., Pomare, C., Ellis, L. A., Long, J. C., Henderson, S. B., Murphy, L. E., & Braithwaite, J. (2021). Patient-reported outcome measures (PROMs): A review of generic and condition‐specific measures and A discussion of trends and issues. *Health Expectations*, *24*(4), 1015–1024. 10.1111/hex.13254 33949755 10.1111/hex.13254 PMC8369118

[7] Weldring, T., & Smith, S. M. (2013). Patient-Reported outcomes (PROs) and Patient-Reported outcome measures (PROMs). *Health Serv Insights*, *6*, 61–68. 10.4137/HSI.S11093PMID: 25114561; PMCID: PMC4089835. 25114561 10.4137/HSI.S11093 PMC4089835

[8] van der Wees, P. J., Verkerk, E. W., Verbiest, M. E., Zuidgeest, M., Bakker, C., Braspenning, J., & van Dulmen, S. A. (2019). Development of a framework with tools to support the selection and implementation of patient-reported outcome measures. *Journal of Patient-Reported Outcomes*, *3*, 1–10. 10.1186/s41687-019-0171-9 31889232 10.1186/s41687-019-0171-9 PMC6937349

[9] Al Sayah, F., Lahtinen, M., Bonsel, G. J., Ohinmaa, A., & Johnson, J. A. (2021). A multi-level approach for the use of routinely collected patient-reported outcome measures (PROMs) data in healthcare systems. *Journal of Patient-Reported Outcomes*, *5*, 1–6. 10.1186/s41687-021-00320-3 34637031 10.1186/s41687-021-00375-1 PMC8511251

[10] Field, J., Holmes, M. M., & Newell, D. (2019). PROMs data: Can it be used to make decisions for individual patients? A narrative review. *Patient Related Outcome Measures*, *10*, 233–241. 10.2147/PROM.S156291 31534379 10.2147/PROM.S156291 PMC6681163

[11] de Jong, K., Conijn, J. M., Gallagher, R. A. V., Reshetnikova, A. S., Heij, M., & Lutz, M. C. (2021). Using progress feedback to improve outcomes and reduce drop-out, treatment duration, and deterioration: A multilevel meta-analysis. *Clinical Psychology Review*, *85*, 102002. 10.1016/j.cpr.2021.102002 33721605 10.1016/j.cpr.2021.102002

[12] Solstad, S. M., Kleiven, G. S., & Moltu, C. (2021). Complexity And potentials of clinical feedback in mental health: An in-depth study of patient processes. *Quality of Life Research*, *30*(11), 3117–3125. 10.1007/s11136-021-02859-6 32556824 10.1007/s11136-020-02550-1 PMC8528773

[13] Rekkers, M., Scheffers, M., van Elburg, A. A., & van Busschbach, J. T. (2021). The protocol for positive body experience (PBE); introducing a psychomotor therapy intervention based on positive body exposure targeting negative body image in eating disorders. *Body Movement and Dance in Psychotherapy*, *16*(4), 252–266. 10.1080/17432979.2021.1968756

[14] Scheffers, M., van Busschbach, J. T., Bosscher, R. J., Aerts, L. C., Wiersma, D., & Schoevers, R. A. (2017). Body image in patients with mental disorders: Characteristics, associations with diagnosis and treatment outcome. *Comprehensive Psychiatry*, *74*, 53–60. 10.1016/j.comppsych.2017.01.002 28095340 10.1016/j.comppsych.2017.01.004

[15] van der Maas, L. C., Köke, A., Pont, M., Bosscher, R. J., Twisk, J. W., Janssen, T. W., & Peters, M. L. (2015). Improving the multidisciplinary treatment of chronic pain by stimulating body awareness: A cluster-randomized trial. *The Clinical Journal of Pain*, *31*(7), 660–669. 10.1097/AJP.0000000000000152 25119509 10.1097/AJP.0000000000000138

[16] Boerhout, C., Swart, M., Voskamp, M., Troquete, N. A., van Busschbach, J. T., & Hoek, H. W. (2017). Aggression regulation in day treatment of eating disorders: Two-centre RCT of a brief body and movement‐oriented intervention. *European Eating Disorders Review*, *25*(1), 52–59. 10.1002/erv.2487 27862660 10.1002/erv.2491

[17] Mehling, W. E., Acree, M., Stewart, A., Silas, J., & Jones, A. (2018). The multidimensional assessment of interoceptive awareness, version 2 (MAIA-2). *PloS One*, *13*(12), e0208034. 10.1371/journal.pone.0208034 30513087 10.1371/journal.pone.0208034 PMC6279042

[18] Humphrey-Murto, S., Varpio, L., Gonsalves, C., & Wood, T. J. (2017). Using consensus group methods such as Delphi and nominal group in medical education research. *Medical Teacher*, *39*(1), 14–19. 10.1080/0142159X.2017.1245856 27841062 10.1080/0142159X.2017.1245856

[19] Mullen, R., Kydd, A., Fleming, A., & McMillan, L. (2021). A practical guide to the systematic application of nominal group technique. *Nurse Researcher*, *29*(1), 14–20. 10.7748/nr.2021.e1798 33629547 10.7748/nr.2021.e1777

[20] Søndergaard, E., Ertmann, R. K., Reventlow, S., & Lykke, K. (2018). Using a modified nominal group technique to develop general practice. *BMC Family Practice*, *19*(1), 117. 10.1186/s12875-018-0804-6 30021508 10.1186/s12875-018-0811-9 PMC6052560

[21] Carless, D., & Douglas, K. (2017). Narrative research. *The Journal of Positive Psychology*, *12*(3), 307–308. 10.1080/17439760.2016.1262611

[22] World Health Organization. (2001). *The international classification of Functioning, disability and health*. Geneva.

[23] World Health Organization. (2024). *International classification of Functioning, disability and health*. Eleventh revision. Geneva.

[24] McMillan, S. S., King, M., & Tully, M. P. (2016). How to use the nominal group and Delphi techniques. *International Journal of Clinical Pharmacy*, *38*(3), 655–662. 10.1007/s11096-016-0257-x 26846316 10.1007/s11096-016-0257-x PMC4909789

[25] Rahimi, S., & Khatooni, M. (2024). Saturation in qualitative research: An evolutionary concept analysis. *International Journal of Nursing Studies Advances*, *6*, 100174. 10.1016/j.ijnsa.2024.100174 38746797 10.1016/j.ijnsa.2024.100174 PMC11080421

[26] Hennink, M., & Kaiser, B. N. (2022). Sample sizes for saturation in qualitative research: A systematic review of empirical tests. *Social Science & Medicine*, *292*, 114523. 10.1016/j.socscimed.2021.114523 34785096 10.1016/j.socscimed.2021.114523

[27] Knott, E., Rao, A. H., Summers, K., et al. (2022). Interviews in the social sciences. *Nat Rev Methods Primers*, *2*, 73. 10.1038/s43586-022-00150-6

[28] Mallett, R., McLean, S., Holden, M. A., Potia, T., Gee, M., & Haywood, K. (2020). Use of the nominal group technique to identify UK stakeholder views of the measures and domains used in the assessment of therapeutic exercise adherence for patients with musculoskeletal disorders. *British Medical Journal Open*, *10*(2), e030956. 10.1136/bmjopen-2019-030956 10.1136/bmjopen-2019-031591 PMC7044886 32075824

[29] Amir, N., Guha, C., Carter, S., & Jauré, A. (2024). Focus groups. In J. E. Edlund, & A. L. Nichols (Eds.), *The Cambridge handbook of research methods and statistics for the social and behavioral sciences* (Vol. 2, pp. 640–664). Cambridge University Press.

[30] Kerr, C., Nixon, A., & Wild, D. (2010). Assessing and demonstrating data saturation in qualitative inquiry supporting patient-reported outcomes research. *Expert Review of Pharmacoeconomics & Outcomes Research*, *10*(3), 269–281. 10.1586/erp.10.30 20545592 10.1586/erp.10.30

[31] Mentimeter (n.d.). [Interactive polling software]. https://www.mentimeter.com

[32] Mehling, W. E., Gopisetty, V., Daubenmier, J., Price, C. J., Hecht, F. M., & Stewart, A. (2009). Body awareness: Construct and self-report measures. *PloS One*, *4*(5), e5614. 10.1371/journal.pone.0005614 19440300 10.1371/journal.pone.0005614 PMC2680990

[33] Brooks-Harris, J. E. (2008). *Integrative multitheoretical psychotherapy*. Houghton Mifflin.

[34] McClelland, M. M., Ponitz, C. C., Messersmith, E., & Tominey, S. (2010). Self-regulation: The integration of cognition and emotion. In W. F. Overton (Vol. Ed.) & R. M. Lerner (Series Ed.), *Handbook of life-span development: Vol. 1. Cognition, biology, and methods* (pp. 509–553). Hoboken, NJ: John Wiley & Sons.

[35] Haeyen, S. (2022). Effects of arts and psychomotor therapies in personality disorders. Developing a treatment guideline based on a systematic review using GRADE. *Frontiers in Psychiatry*, *13*, 878866. 10.3389/fpsyt.2022.878866 35782411 10.3389/fpsyt.2022.878866 PMC9243752

[36] Röhricht, F. (2015). Body psychotherapy for the treatment of severe mental disorders–an overview. *Body Movement and Dance in Psychotherapy*, *10*(1), 51–67. 10.1080/17432979.2014.978899

[37] Rosendahl, S., Sattel, H., & Lahmann, C. (2021). Effectiveness of body psychotherapy. A systematic review and meta-analysis. *Frontiers in Psychiatry*, *12*, 709798. 10.3389/fpsyt.2021.709798 34566712 10.3389/fpsyt.2021.709798 PMC8458738

[38] Dong, Y., Zhang, X., Zhao, R., Cao, L., Kuang, X., & Yao, J. (2024). The effects of mind-body exercise on anxiety and depression in older adults: a systematic review and network meta-analysis. *Frontiers in Psychiatry, 15*, 1305295. 10.3389/fpsyt.2024.1305295 10.3389/fpsyt.2024.1305295 PMC10879425 38384592

[39] van de Kamp, M. M., Scheffers, M., Hatzmann, J., Emck, C., Cuijpers, P., & Beek, P. J. (2019). Body- and movement‐oriented interventions for posttraumatic stress disorder: A systematic review and meta‐analysis. *Journal of Traumatic Stress, 32*(6), 967–976. 10.1002/jts.22467. 10.1002/jts.22465 PMC6973294 31658401

[40] van de Kamp, M. M., Scheffers, M., Emck, C., Fokker, T. J., Hatzmann, J., Cuijpers, P., & Beek, P. J. (2023). Body- and movement‐oriented interventions for posttraumatic stress disorder: An updated systematic review and meta‐analysis. *Journal of Traumatic Stress*, *36*(5), 835–848. 10.1002/jts.22912 37702005 10.1002/jts.22968

[41] Hernandez-Posadas, A., Lommen, M. J. J., de la Rosa Gomez, A., Bouman, T. K., Mancilla-Diaz, J. M., & Gonzalez, D. P., A (2023). Transdiagnostic factors in symptoms of depression and post-traumatic stress: A systematic review. *Current Psychology*, 1–16. 10.1007/s12144-023-04815-4 10.1007/s12144-023-04792-x PMC10226442 37359653

[42] Krueger, R. F., & Eaton, N. R. (2015). Transdiagnostic factors of mental disorders. *World Psychiatry*, *14*(1), 27. 10.1002/wps.20175 25655146 10.1002/wps.20175 PMC4329885

[43] McEvoy, P. M. (2022). 11.09 - Transdiagnostic Approaches to Mental Disorders. In G. J. G. Asmundson (Ed.), *Comprehensive Clinical Psychology (Second Edition)* (pp. 112–124). Elsevier. 10.1016/B978-0-12-818697-8.00111-2

[44] Scheffers, M., van Duijn, M. A. J., Beldman, M., Bosscher, R. J., van Busschbach, J. T., & Schoevers, R. A. (2019). Body attitude, body satisfaction and body awareness in a clinical group of depressed patients: An observational study on the associations with depression severity and the influence of treatment. *Journal of Affective Disorders*, *242*, 22–28. 10.1016/j.jad.2018.08.066 30170235 10.1016/j.jad.2018.08.074

[45] van de Kamp, M. M., Emck, C., Scheffers, M., Hoven, M., Cuijpers, P., & Beek, P. J. (2024). Psychomotor therapy for posttraumatic stress disorder. *Body, Movement and Dance in Psychotherapy*, 1–19. 10.1080/17432979.2024.2433487

[46] Kalisvaart, J. B. (2019). *Body-relatedness in somatic symptom disorder* (Doctoral dissertation, Utrecht University). 10.33612/diss.2019.kalisvaart

[47] Aldao, A., & Nolen-Hoeksema, S. (2010). Specificity of cognitive emotion regulation strategies: A transdiagnostic examination. *Behaviour Research and Therapy*, *48*(10), 974–983. 10.1016/j.brat.2010.06.002 20591413 10.1016/j.brat.2010.06.002

[48] Sloan, E., Hall, K., Moulding, R., Bryce, S., Mildred, H., & Staiger, P. K. (2017). Emotion regulation as a transdiagnostic treatment construct across anxiety, depression, substance, eating and borderline personality disorders: A systematic review. *Clinical Psychology Review*, *57*, 141–163. 10.1016/j.cpr.2017.09.002 28941927 10.1016/j.cpr.2017.09.002

[49] Conway, C. C., Hammen, C., & Brennan, P. A. (2012). Expanding stress generation theory: Test of a transdiagnostic model. *Journal of Abnormal Psychology*, *121*(3), 754–766. 10.1037/a0027457 22428789 10.1037/a0027457 PMC4830479

[50] Zimmer-Gembeck, M. J., Skinner, E. A., Modecki, K. L., Webb, H. J., Gardner, A. A., Hawes, T., & Rapee, R. M. (2018). The self-perception of flexible coping with stress: A new measure and relations with emotional adjustment. *Cogent Psychology*, *5*(1), 1537908. 10.1080/23311908.2018.1537908

[51] Wilson, S., Stroud, C. B., & Durbin, C. E. (2017). Interpersonal dysfunction in personality disorders: A meta-analytic review. *Psychological Bulletin*, *143*(7), 677–734. 10.1037/bul0000090 28447827 10.1037/bul0000101 PMC5507693

[52] Sondergaard, M., & Fisher, A. G. (2012). Sensitivity of the evaluation of social interaction measures among people with and without neurologic or psychiatric disorders. *American Journal of Occupational Therapy*, *66*(3), 356–362. 10.5014/ajot.2012.003509 10.5014/ajot.2012.003582 22549601

[53] Derntl, B., & Habel, U. (2011). Deficits in social cognition: A marker for psychiatric disorders? *European Archives of Psychiatry and Clinical Neuroscience*, *261*(Suppl 2), S145–S149. 10.1007/s00406-011-0244-0 21863344 10.1007/s00406-011-0244-0

[54] Shankman, S. A., Katz, A. C., DeLizza, A. A., Sarapas, C., Gorka, S. M., & Campbell, M. L. (2014). The Different Facets of Anhedonia and Their Associations with Different Psychopathologies. In M. S. Ritsner (Ed.), *Anhedonia: A Comprehensive Handbook Volume I: Conceptual Issues And Neurobiological Advances* (pp. 3–22). Springer Netherlands. 10.1007/978-94-017-8591-4_1

[55] Semple, S. J., Patterson, T. L., Shaw, W. S., Grant, I., Moscona, S., & Jeste, D. V. (1999). Self-perceived interpersonal competence in older schizophrenia patients: The role of patient characteristics and psychosocial factors. *Acta Psychiatrica Scandinavica*, *100*(2), 126–135. 10.1111/j.1600-0447.1999.tb10620.x 10480198 10.1111/j.1600-0447.1999.tb10833.x

[56] Uhrlass, D. J., Schofield, C. A., Coles, M. E., & Gibb, B. E. (2009). Self-perceived competence and prospective changes in symptoms of depression and social anxiety. *Journal of Behavior Therapy and Experimental Psychiatry*, *40*(2), 329–337. 10.1016/j.jbtep.2009.01.001 19168174 10.1016/j.jbtep.2009.01.001 PMC4113081

[57] Barch, D. M., Pagliaccio, D., Luking, K., Moran, E. K., & Culbreth, A. J. (2019). Pathways to motivational impairments in psychopathology: Common versus unique elements across domains. In M. Neta & I. J. Haas (Eds.), *Emotion in the Mind and Body* (pp. 121–160). Springer Nature Switzerland AG. 10.1007/978-3-030-27473-3_5

[58] Van der Meijden, H., & Bosscher, R. (2007). *Psychomotorische therapie voor mensen met chronische pijn [Psychomotor therapy for people with chronic pain]*. Hogeschool Windesheim.

[59] Emck, C., Hammink, M. N., & Bosscher, R. J. (2007). *Psychomotorische diagnostiek en indicatiestelling voor kinderen van 6 tot 12 jaar* [Psychomotor diagnosis and indications for psychomotor therapy for children aged 6 to 12 years ]. ‘t Web.

[60] Vahedian-Shahroodi, M., Mansourzadeh, A., Shariat Moghani, S., & Saeidi, M. (2023). Using the nominal group technique in group Decision-Making: A review. *Medical Education Bulletin*, *4*(4), 837–845. 10.22034/meb.2024.434656.1090

