# Correction: Identifying key outcome domains with underlying specific patient-reported outcomes for psychomotor therapy in mental health care in the Netherlands: a multi-phased qualitative study

**DOI:** 10.1007/s11136-026-04228-6

**Published:** 2026-04-10

**Authors:** Albertine de Haan, Janet Moeijes, Mia Scheffers, Philip van der Wees

**Affiliations:** 1 Department of Human Movement and Education, Windesheim University of Applied Sciences, Zwolle, The Netherlands; 2 Radboud University Medical Center, IQ Health and Department of Rehabilitation, Nijmegen, The Netherlands

**Correction to: Quality of Life Research (2026) 35:49** 10.1007/s11136-025-04119-2

In this article, the number of the educational program's of the focus group participants is incorrect. The incorrect and corrected table 1 are provided in this correction.

**Incorrect Table 1**

**Table 1:** Characteristics of psychomotor professionals (*n* = 53)^1^ in sub-study (i)

	Group interviews (*n* = 31)	Focus groups (*n* = 23)	Consensus meeting (*n* = 6)
*Gender*			
Male	–	10	3
Female	–	13	3
*Profession* ^2^			
Psychomotor therapist	–	18	2
Research	–	9	2
Education	–	3	4
*Educational program* ^3^			
Bachelor Windesheim University	20	20	1
Master Windesheim University	6	6	3
Bachelor HAN University	5	5	1

^1^The total number of psychomotor professionals is less than the sum of all groups due to participation in more than one group of some psychomotor professionals

^2^The total number of professions exceeds the total number of psychomotor professionals due to the various professions of some psychomotor professionals

^3^The total number of educational programs exceeds the total number of psychomotor professionals due to the various educational activities of some psychomotor professionals

**Correct Table 1**

**Table 1:** Characteristics of psychomotor professionals (*n* = 53)^1^ in sub-study (i)

	Group interviews (*n* = 31)	Focus groups (*n* = 23)	Consensus meeting (*n* = 6)
*Gender*			
Male	–	10	3
Female	–	13	3
*Profession*^2^			
Psychomotor therapist	–	18	2
Research	–	9	2
Education	–	4	4
*Educational program*^3^			
Bachelor Windesheim University	20	2	1
Master Windesheim University	6	3	3
Bachelor HAN University	5	0	1

^1^The total number of psychomotor professionals is less than the sum of all groups due to participation in more than one group of some psychomotor professionals

^2^The total number of professions exceeds the total number of psychomotor professionals due to the various professions of some psychomotor professionals

^3^The total number of educational programs exceeds the total number of psychomotor professionals due to the various educational activities of some psychomotor professionals

The original article has been corrected.

