# Supplementary material for: Identifying key outcome domains with underlying specific patient-reported outcomes for psychomotor therapy in mental health care in the Netherlands: a multi-phased qualitative study
Source: Qual Life Res. 2026 Jan 21;35(2):49. doi: 10.1007/s11136-025-04119-2 (PMC12823721; doi:10.1007/s11136-025-04119-2)
Supplement: Supplementary file 1 — Supplementary Material 1 [file 11136_2025_4119_MOESM1_ESM.docx]

**Title**

Identifying key outcome domains with underlying specific patient-reported outcomes for psychomotor therapy in mental health care in the Netherlands: a multi-phased qualitative study

**Journal**

Quality of Life Research

**Authors**

**Albertine de Haan^1,2^**

Corresponding author

E-mail address: [Albertine.deHaan@radboudumc.nl](mailto:Albertine.deHaan@radboudumc.nl)

ORCID: [0009-0009-4990-1428](https://orcid.org/0009-0009-4990-1428)

**Dr. Janet Moeijes^1^**

ORCID: 0000-0002-1491-0246

**Dr. Mia Scheffers^1^**

ORCID: [0000-0003-0469-1569](https://orcid.org/0000-0003-0469-1569)

**Prof. Philip van der Wees^2^**

ORCID: [0000-0003-2881-5159](https://orcid.org/0000-0003-2881-5159)

^1^Department of Human Movement and Education, Windesheim University of Applied

Sciences, Zwolle, the Netherlands

^2^Radboud University Medical Center, IQ Health and Department of Rehabilitation, Nijmegen, the Netherlands

**Online resource 1**

**ICF framework**

To enhance the comprehensiveness and clarity of the five most relevant outcome domains with underlying PROs for psychomotor therapy, the domains were categorised using the International Classification of Functioning, Disability and Health (ICF) developed by the World Health Organization [1,2].

**Rationale**

The ICF is an internationally recognised framework for describing and assessing functioning from a biopsychosocial perspective [1], which is particularly relevant in mental health care, where disorders are shaped by complex interactions between biological, psychological, and socio-environmental factors [3]. In addition, the ICF has been implemented in clinical settings in the Netherlands for outcome evaluation [4] underscoring its suitability for linking study outcomes and facilitating communication among healthcare professionals. By linking the five most relevant outcome domains and underlying specific PROs for psychomotor therapy with the ICF-framework, the key domains can be presented in a structured and comprehensive manner, highlighting the aspects of health most likely to be influenced by psychomotor therapy from the patient’s perspective.

**Methods**

The five most relevant outcome domains for psychomotor therapy in adult mental health care were systematically mapped onto the ICF framework, at the level of components, domains and category titles using the linking rules of the ICF [2]. This mapping followed the available ICF Core Sets for mental health [5] ensuring consistency with the international mental health care context and enhancing the applicability of findings to both clinical practice and interdisciplinary communication.

Appropriate ICF codes, along with their associated components, domains and category titles, representing the five most relevant outcome domains and corresponding PROs for psychomotor therapy, were identified by two researchers (AH, JM) until consensus was reached. In cases of disagreement, a third researcher (MS) was consulted. All entries in the ICF codebook were systematically reviewed.

**Results**

The five most relevant outcome domains for psychomotor therapy are categorised in the ICF framework in Table 1.

**Table 1**

*The five most relevant outcome domains for psychomotor therapy linked with the ICF framework*

| **Outcome domains for psychomotor therapy** | **ICF component** | **ICF domains** | **ICF category titles** |
| --- | --- | --- | --- |
| Body experience | Body functions | Mental functions | Orientation functions  Global psychosocial functions  Temperament and personality functions  Energy and drive functions  Attention functions  Emotional functions  Perceptual functions  Thought functions  Higher-level cognitive functions  Experience of self and time functions |
|  |  | Sensory functions and pain | Additional sensory functions |
|  | Activities and participation | Learning and applying knowledge | Purposeful sensory experiences  Handling stress and other psychological demands  Caring for body parts  Looking after one’s health |
| Movement experience | Body functions | Mental functions | Orientation functions  Temperament and personality functions  Energy and drive functions  Psychomotor functions  Thought functions  Higher-level cognitive functions  Experience of self and time functions |
|  |  | Sensory functions and pain | Additional sensory functions |
|  | Activities and participation | Mobility | Walking  Moving around |
|  |  | Community, social and civic life | Recreation and leisure |
| Emotion regulation | Body functions | Mental functions | Orientation functions  Global psychosocial functions  Temperament and personality functions  Energy and drive function  Attention functions  Psychomotor functions  Emotional functions  Perceptual functions  Thought functions  Higher-level thought function  Mental functions of language  Experience of self and time functions |
|  |  | Sensory functions and pain | Additional sensory functions |
|  | Activities and participation | Learning and applying knowledge | Focusing attention  Solving problems  Making decisions |
|  |  | General tasks and demands | Handling stress and other psychological demands |
| Social interaction | Body functions | Mental functions | Orientation functions  Global psychosocial functions  Temperament and personality functions  Attention functions  Emotional functions  Perceptual functions  Thought functions  Higher-level cognitive functions  Mental functions of language  Experience of self and time functions |
|  | Activities and participation | Learning and applying knowledge | Purposeful sensory experiences  Focusing attention  Solving problems  Making decisions |
|  |  | General tasks and demands | Undertaking a single task  Undertaking multiple tasks  Handling stress and other psychological demands |
|  |  | Communication | Communicating with - receiving - spoken messages  Communicating with - receiving - nonverbal messages  Speaking  Producing nonverbal messages  Conversation  Discussion |
|  |  | Interpersonal interactions and relationships | Basic interpersonal interactions  Complex interpersonal interactions  Relating with strangers  Formal relationships  Informal social relationships  Intimate relationships |
| Stress regulation | Body functions | Mental functions | Temperament and personality functions  Attention functions  Emotional functions  Thought functions  Experience of self and time |
|  |  | Sensory functions and pain | Additional sensory functions |
|  | Activities and participation | Learning and applying knowledge | Purposeful sensory experiences  Solving problems  Making decisions |
|  |  | General tasks and demands | Handling stress and other psychological demands |

Certain domains for psychomotor therapy are coded using multiple codes due to the absence of a single, representative code. However, it is essential to consider domains for psychomotor therapy as a whole to maintain their conceptual integrity.

**References**

1. World Health Organization (2001). The International Classification of Functioning, Disability and Health. Geneva, WHO.
2. World Health Organization (2024). International Classification of Functioning, Disability and Health. Eleventh revision. Geneva, WHO.
3. Porter, R.J. (2020) The biopsychosocial model in mental health. *Australian & New Zealand Journal of Psychiatry*, 54(8):773-774. doi:[10.1177/0004867420944464](https://doi.org/10.1177/0004867420944464)
4. Leonardi, M., Lee, H., Kostanjsek, N., Fornari, A., Raggi, A., Martinuzzi, A., Yáñez, M., Almborg, A. H., Fresk, M., Besstrashnova, Y., Shoshmin, A., Castro, S. S., Cordeiro, E. S., Cuenot, M., Haas, C., Maart, S., Maribo, T., Miller, J., Mukaino, M., Snyman, S., … Kraus de Camargo, O. (2022). 20 Years of ICF-International Classification of Functioning, Disability and Health: Uses and Applications around the World. *International journal of environmental research and public health*, *19*(18), 11321. <https://doi.org/10.3390/ijerph191811321>
5. ICF Research Branch. (w.d.). *ICF Core Sets – Diverse situations*. Retrieved September 22, 2025, from [https://www.icf-research-branch.org/icf-core-sets/category/11-diversesituations](https://www.icf-research-branch.org/icf-core-sets/category/11-diversesituations?utm_source=chatgpt.com)
