# Supplementary material for: Identifying key outcome domains with underlying specific patient-reported outcomes for psychomotor therapy in mental health care in the Netherlands: a multi-phased qualitative study
Source: Qual Life Res. 2026 Jan 21;35(2):49. doi: 10.1007/s11136-025-04119-2 (PMC12823721; doi:10.1007/s11136-025-04119-2)
Supplement: Supplementary file 2 — Supplementary Material 2 [file 11136_2025_4119_MOESM2_ESM.pdf]

Title

Identifying key outcome domains with underlying specific patient-reported outcomes for psychomotor therapy in mental health care in the Netherlands: a multi-phased qualitative study

Journal

Quality of Life Research

Authors
Albertine de Haan[1,2]
Corresponding author
E-mail address: Albertine.deHaan@radboudumc.nl
ORCID: 0009-0009-4990-1428
Dr. Janet Moeijes[1]
ORCID: 0000-0002-1491-0246
Dr. Mia Scheffers[1]
ORCID: 0000-0003-0469-1569
Prof. Philip van der Wees[2]
ORCID: 0000-0003-2881-5159

[1]Department of Human Movement and Education, Windesheim University of Applied Sciences, Zwolle, the Netherlands
[2]Radboud University Medical Center, IQ Health and Department of Rehabilitation, Nijmegen, the Netherlands

Online resource 2

Methods

The methodology of the study is presented in detail below, thereby promoting both transparency and the interpretability of the findings.

Design and setting
This study, conducted from September 2019 to February 2020, employed a qualitative multi-phased approach. It consisted of three sub-studies:

(i) the identification and prioritisation of the most relevant outcome domains with underlying specific PROs for psychomotor therapy from the perspective of psychomotor professionals with the use of a modified Nominal Group Technique in an adapted serial design (NGT) [1-3];

(ii) the identification and prioritisation of the most relevant outcome domains with underlying specific PROs for psychomotor therapy from the perspective of patients using a narrative approach [4] in focus groups;

and,

(iii) the synthesis of the priority of both selections, resulting in the five most relevant outcome domains with underlying specific PROs for psychomotor therapy.

Figure 1 shows the steps in the sub-studies. Each step in the figure shows the procedures performed and the corresponding results.

Figure 1

Flowchart of sub-studies (i)–(iii) depicting each step with the procedure performed, and the corresponding results

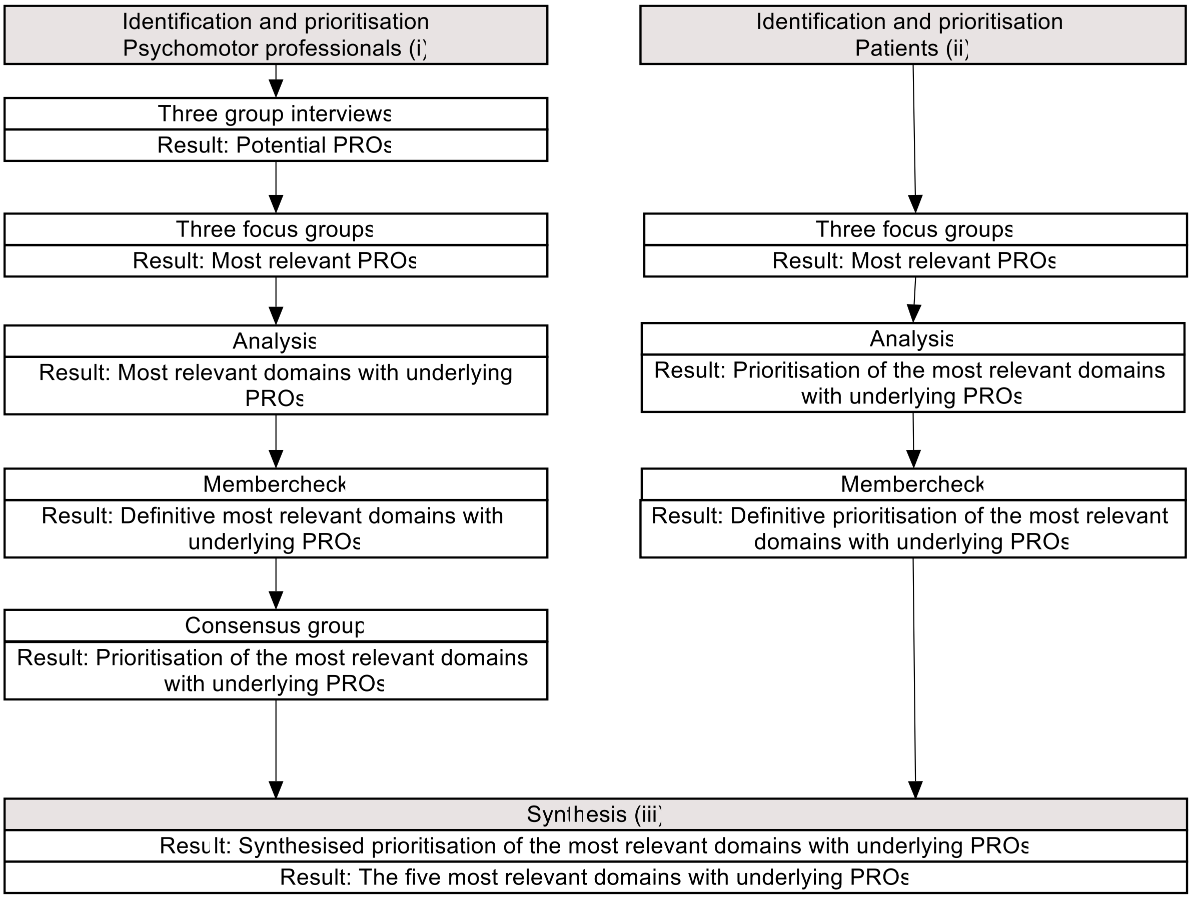

Note: Made with ClickCharts NCH Software

Research team

The research team comprised of six people. A professor with expertise in psychiatry and psychomotor therapy research supervised the study (JB). Furthermore, a team of researchers which comprised an associate professor (MS) with senior expertise in psychomotor therapy, research, outcome measures, and group dynamics, a senior researcher (JM) with senior expertise in psychomotor therapy research and group dynamics, and a junior researcher (AH) with senior expertise in psychomotor therapy and group dynamics executed the study. Two junior researchers with senior expertise in psychomotor therapy (IN, MR), assisted and gave advice.

Sub-study (i): Identification and prioritisation of the most relevant outcome domains with underlying specific PROs for psychomotor therapy by psychomotor professionals

A classical NGT face-to-face group meeting consists of solo idea generation, round-robin feedback on ideas, clarification, and voting to prioritise ideas [5]. This sub-study used a

modified NGT to include a diverse representation of experts in psychomotor therapy and research to identify and prioritise the most relevant domains with underlying specific PROs for psychomotor therapy from the perspective of psychomotor professionals. The modified NGT was applied across three group interviews (idea generation of PROs), three focus groups (individual selection of the most relevant PROs, discussion on the first selection, and final individual selection), and one consensus group meeting (voting to prioritise the most relevant domains with underlying PROs, which were compiled after the analysis of the focus groups). Due to the inclusion of the total number of groups and diverse participants in the study, data and saturation [6,7] were anticipated. All meetings were conducted face-to-face, and the collection of data was note- and photograph-based.

Procedure participants
The executive researchers (AH, JM, MS) played a key role in the study, inviting all possible participants via phone or email and ensuring their participation. They also applied the pre-elicitation technique, sending an invitation email to the participants that outlined the research objective, research question, and purpose of the interview or meeting [8]. At the start of each meeting, these were shortly repeated. Their efforts were instrumental in ensuring the smooth running of the study and the active participation of the selected participants.

Group interviews
In three group interviews, semi-structured interviews conducted with several people at the same time [9], we used the first step of the modified NGT to generate potential PROs for psychomotor therapy in adult mental healthcare.

Participants
The three groups consisted of lecturers (mostly trained as psychomotor therapists) of all initial and master's educational programs in psychomotor therapy in the Netherlands, because of their different and extensive knowledge of PROs in psychomotor therapy. All lecturers were invited to participate in the group interviews. The interviews were conducted during an existing team meeting of the respective education program to include the maximum number of participants. Participation was based on availability.
Lecturers were affiliated with the bachelor's degree (group 1; n=20) and master's degree (group 2; n=6) programs in psychomotor therapy at Windesheim University of Applied Sciences, Zwolle and the bachelor's degree (group 3; n=5) program at HAN University of Applied Sciences, Nijmegen.

Data collection
In 60-minute group interviews, participants were asked to identify potential PROs for psychomotor therapy in adult mental health care and to elaborate on what each PRO entailed. One researcher (MS) conducted the interviews using an interview guide while two researchers (JM, AH) took notes. Subsequently, the executive researchers held a collective debriefing.

Data analysis
After conducting three group interviews, a list was compiled of all the mentioned PROs. Outcomes that were not considered PROs (objective measures, i.e. physical fitness) were

excluded from this list [10,11]. Finally, a list of potential PROs for psychomotor therapy in adult mental healthcare was compiled.

Focus groups

In three focus groups, guided group discussions to explore participants' perspectives through interaction [12]. The second step of the modified NGT was used to select the most relevant PROs for psychomotor therapy.

Participants

Three distinct focus groups were selected through the purposive sampling of different psychomotor professionals to ensure diverse perspectives. Participants were researchers in psychomotor therapy of the research group Human Movement, Health, and Well-being at Windesheim University of Applied Sciences Zwolle (group 1; n=7; all researchers (n=16) affiliated at the research group were invited; participation was based on availability), psychomotor therapists from the University Centre of Psychiatry (UCP) at University Medical Centre Groningen (UMCG; group 2; n=6; all therapists were invited (n=7); participation was based on availability), and a group of senior psychomotor therapists selected through quota sampling [31] based on expertise in different psychopathology (group 3; n=10; 22 eligible participants were invited based on their seniority, expertise with one of more psychiatric disorders and membership of a working group of that specific psychiatric disorder; participants only reclined the invitation based on availability). There was one person who participated in both the group interviews and the focus groups. This person had senior expertise in psychomotor therapy, was a lecturer and a researcher, and had extensive expertise in the field of psychomotor therapy.

Data collection

During three 90-minute focus groups, participants were provided with sets of index cards, each displaying a single PRO previously identified in the group interviews. In the first phase, participants independently selected the five PROs they considered most relevant, without discussion, by choosing the corresponding index cards. Participants were also allowed to introduce new PROs if they identified gaps. In the second phase, participants shared their selections in a group discussion to explore underlying rationales. In the final phase, participants were asked to reconsider their choices in light of the discussion and confirm their five most relevant PROs for psychomotor therapy.
One researcher (MS) moderated the focus groups using a guide, and two researchers (JM, AH) co-moderated and took notes. The executive researchers held a collective debriefing after the meeting. The selected index cards from both selection rounds were photographed.

Data analysis

After each focus group, newly mentioned PROs were added to the index cards for the next meeting. Although a single new theme was identified in the concluding focus group, the research team determined that data saturation had been reached, given that no further meaningful insights were anticipated.
Following completion of the three focus groups, only the participants' final selections of PROs were analysed. PROs were clustered and assigned to outcome domains based on group

interview and focus group notes, and the literature by two researchers, who reached consensus through discussion (AH, JM). If a domain comprised multiple subdomains, this would be documented in the reports and results produced within the current sub-study and sub-study (iii). The categorisation of subdomains was likewise conducted collaboratively by these researchers; in cases of disagreement, a third researcher (MS) was consulted.
Finally, a report of the outcome domains with underlying specific PROs for psychomotor therapy was compiled and sent to all participants of the group interviews and focus groups for a member check. The participants' feedback was processed (AH, JM, in case of non-agreement MS was consulted), resulting in a definitive report.

Consensus group meeting

In a consensus group meeting, the third and last step of the modified NGT was used to prioritise the most relevant outcome domains with underlying specific PROs for psychomotor therapy.

Participants
A 90-minute consensus group meeting (n=6) included participants from the previous group interviews and focus group meetings to ensure representation from the groups that participated earlier. The consensus group was invited by the research team, based on the willingness and availability of participants from the group interviews and focus groups, using quota sampling. Two lecturers, two psychomotor therapists and two researchers participated.

Data collection
A week before the meeting, participants received the definitive report of the results of the focus groups. The report was presented and discussed. Participants then individually, subjectively, and anonymously ranked the domains and underlying PROs using the digital tool Mentimeter [13]. Each participant assigned the highest rank to the domain they considered most relevant, followed by successive ranks in descending order of relevance, with the lowest rank assigned to the domain judged least relevant. Mentimeter automatically calculated the mean ranking for each domain across participants and subsequently generated an aggregated list. In this list, the domain with the highest mean rank reflected greater perceived relevance as determined by the participants and received the highest ranking number.
In a subsequent step, participants distributed a total of 100 points (in total 600 points by all participants) across all domains using Mentimeter. A higher number of points indicated greater perceived relevance, whereas fewer or zero points reflected lesser relevance. Mentimeter then produced an aggregated list ordered from the highest to lowest points received, with the domain receiving the most points at the top of the list, being perceived as the most relevant, receiving the highest rating number.
For the analysis, the ranking and rating numbers of the domains from both lists were summed, with lower totals indicating higher relevance. In the event of ties, the number of times a domain was selected in the focus group determined its priority, with more frequently selected domains considered more relevant. Then, the researchers produced an aggregated list of the domains in order of relevance.
Finally, the participants rated their percentage of agreement (0-100%) with the order of relevance of the domains, using Mentimeter independently and subjectively. Consensus was considered at a mean of 70% agreement [2]. In case of less than 70% agreement, a group

discussion, rating, ranking and consensus rating were repeated, until 70% agreement was reached.
One researcher (MS) moderated the meetings, and two researchers (AH, JM) co-moderated and took notes. The executive researchers held a collective debriefing after the meeting.

Data analysis
The definite report was revised if PROs were assigned to a different domain based on the notes from the discussion and overall agreement of the participants. A list was compiled (AH, JM), presenting the prioritisation of the most relevant outcome domains with their underlying specific PROs for psychomotor therapy, as identified by psychomotor professionals.

Sub-study (ii): Identification and prioritisation of the most relevant outcome domains with underlying specific PROs for psychomotor therapy by patients
To identify and prioritise the most relevant domains and underlying specific PROs for psychomotor therapy from the perspective of patients, three 90-minute face-to-face focus groups with a narrative approach were conducted.

Participants
Participants were recruited in collaboration with the mental health institute Altrecht in Utrecht (group 1; n=12) and the UCP of the UMCG Groningen (group 2; n=8 and group 3; n=7). Sites did not report on refusal rates. Participants at the UCP were assigned to a focus group based on their availability. Purposive sampling was used to invite patients with different psychiatric disorders who had undergone group- and/or individual psychomotor treatment. Inclusion criteria were the ability to engage in a group conversation and be older than 18 years of age.
The psychomotor therapists working at Altrecht and the UCP did the selection and invitations by phone or email. Interested (former) patients were sent an informed consent form with a week's reflection period. Participants were briefed about the research question and the focus group's objective through email and at the start of the focus group. The focus groups took place in the respective treatment institutes.

Data collection
In the patient focus groups, each participant was asked to identify and select their three most relevant PROs from their individual experience, rather than five, as in the professional group, since patients primarily focus on their own experiences. In contrast, psychomotor professionals draw on broader clinical experience across diverse patients and therefore had the opportunity to incorporate their knowledge in those five PROs.
Participants began by writing down their individual three most relevant PROs on an index card in a silent round. Then, they elaborated on their experiences regarding these PROs, first individually and subsequently through a group discussion.
One researcher (MS/AH) moderated the meetings, and one or two researchers (AH/JM/IN) co-moderated and took notes. The PROs mentioned by the participants were written on a whiteboard visible to all participants. The written cards and the whiteboard notes were photographed. The executive researchers held a collective debriefing after each focus group.

Data analysis
Saturation of identifying PROs was anticipated due to the inclusion of a sufficient number of groups and diverse participants in the study [6]. Data were analysed (AH, JM; in case of non-agreement MS was consulted) and PROs were categorised in domains after each group meeting, reaching agreement. Prioritisation of domains with underlying specific PROs for psychomotor therapy was based on the number of selected PROs in a domain, with higher prevalence indicating higher prioritisation. A report of the prioritised outcome domains with underlying specific PROs for psychomotor therapy was sent to all participants for a member check. The participants' feedback was processed, resulting in a definitive report on the prioritisation of the most relevant domains with underlying specific PROs for psychomotor therapy from the patient's perspective.

Sub-study (iii) The synthesis of the priority of sub-study (i) and sub-study (ii), resulting in the five most relevant outcome domains with underlying specific PROs for psychomotor therapy

To synthesise the prioritisations from the perspective of psychomotor professionals (i) and patients (ii), and to identify the five most relevant outcome domains, the research team held a 90-minute meeting.

Participants
All members of the research team (n=6) participated in the meeting.

Data analysis
First, the executing researchers presented the results. Domains from both studies were merged where appropriate. Then, the prioritisations of the most relevant domains of both psychomotor professionals and patients were listed in order of relevance. The orders of relevance of the domains from both lists were summed and divided by two (or one if a domain was only mentioned by one group) to calculate the mean sum of the orders of relevance, with lower totals indicating higher relevance. In the event of ties, the number of selections of the domains in the focus groups determined their priority, with more frequently selected domains considered more relevant. Finally, the underlying PROs of both the psychomotor professionals' and the patients' domains were added to the corresponding domain. As a result, the synthesised prioritisation of the most relevant domains with underlying specific PROs for psychomotor therapy was compiled. The first five domains of this synthesised prioritisation were considered to be the five most relevant outcome domains with underlying specific PROs for psychomotor therapy in adult mental health care.
One researcher led the discussion (MS), while two researchers served as co-moderators (AH, JM) and took notes. The executive researchers held a collective debriefing after the synthesis meeting.

References

1. Humphrey-Murto, S., Varpio, L., Gonsalves, C., & Wood, T. J. (2017). Using consensus group methods such as Delphi and Nominal Group in medical education research. *Medical Teacher*, 39(1), 14–19. https://doi.org/10.1080/0142159X.2017.1245856
2. Mullen, R., Kydd, A., Fleming, A., & McMillan, L. (2021). A practical guide to the systematic application of nominal group technique. *Nurse Researcher*, 29(1), 14-20. https://doi.org/10.7748/nr.2021.e1798
3. Søndergaard, E., Ertmann, R. K., Reventlow, S., & Lykke, K. (2018). Using a modified nominal group technique to develop general practice. *BMC Family Practice*, 19(1), 117. https://doi.org/10.1186/s12875-018-0804-6
4. Carless, D., & Douglas, K. (2017). Narrative research. *The Journal of Positive Psychology*, 12(3), 307-308. https://doi.org/10.1080/17439760.2016.1262611
5. McMillan, S. S., King, M., & Tully, M. P. (2016). How to use the nominal group and Delphi techniques. *International Journal of Clinical Pharmacy*, 38(3), 655–662. https://doi.org/10.1007/s11096-016-0257-x
6. Rahimi, S., & Khatooni, M. (2024). Saturation in qualitative research: An evolutionary concept analysis. *International Journal of Nursing Studies Advances*, 6, 100174. https://doi.org/10.1016/j.ijnsa.2024.100174
7. Hennink, M., & Kaiser, B. N. (2022). Sample sizes for saturation in qualitative research: A systematic review of empirical tests. *Social Science & Medicine*, 292, 114523. https://doi.org/10.1016/j.socscimed.2021.114523
8. McMillan, S. S., Kelly, F., Sav, A., Kendall, E., King, M. A., Whitty, J. A., & Wheeler, A. J. (2014). Using the nominal group technique: how to analyse across multiple groups. *Health Services and Outcomes Research Methodology*, 14, 92-108. https://doi.org/10.1007/s10742-014-0121-1
9. Knott, E., Rao, A.H., Summers, K. *et al.* Interviews in the social sciences. *Nat Rev Methods Primers* **2**, 73 (2022). https://doi.org/10.1038/s43586-022-00150-6
10. Weldring T, Smith SM. Patient-Reported Outcomes (PROs) and Patient-Reported Outcome Measures (PROMs). Health Serv Insights. 2013 Aug 4;6:61-8. doi: 10.4137/HSI.S11093. PMID: 25114561; PMCID: PMC4089835.
11. Mallett, R., McLean, S., Holden, M. A., Potia, T., Gee, M., & Haywood, K. (2020). Use of the nominal group technique to identify UK stakeholder views of the measures and domains used in the assessment of therapeutic exercise adherence for patients with musculoskeletal disorders. *BMJ Open*, 10(2), e030956. https://doi.org/10.1136/bmjopen-2019-030956
12. Amir, N., Guha, C., Carter, S., & Jauré, A. (2024). Focus groups. In J. E. Edlund & A. L. Nichols (Eds.), *The Cambridge Handbook of Research Methods and Statistics for the Social and Behavioral Sciences* (Vol. 2, pp. 640-664). Cambridge University Press.
13. Mentimeter. (n.d.). [Interactive polling software]. https://www.mentimeter.com
